# Data on pure tin by Positron Annihilation Lifetime Spectroscopy (PALS) acquired with a semi-analog/digital setup using DDRS4PALS

**DOI:** 10.1016/j.dib.2018.11.121

**Published:** 2018-11-28

**Authors:** Danny Petschke, Ricardo Helm, Torsten E.M. Staab

**Affiliations:** University Wuerzburg, Department of Chemistry and Pharmacy, LCTM Roentgenring 11, D-97070 Wuerzburg, Germany

## Abstract

Positron annihilation lifetime spectroscopy (PALS) provides a powerful technique for non-destructive microstructure investigations in a broad field of material classes such as metals, semiconductors, polymers or porous glasses. Even though this method is well established for more than five decades, no proper standardization for the used setup configuration and subsequent data processing exists. Eventually, this could lead to an insufficiency of data reproducibility and avoidable deviations.

Here we present experimentally obtained and simulated data of positron lifetime spectra at various statistics measured on pure tin (4N-Sn) by using a semi-analog/digital setup, where the digital section consists of the DRS4 evaluation board, “Design and performance of the 6 GHz waveform digitizing chip DRS4” [1]. The analog section consists of nuclear instrument modules (NIM), which externally trigger the DRS4 evaluation board to reduce the digitization and, thus, increase the acquisition efficiency. For the experimentally obtained lifetime spectra, ^22^Na sealed in Kapton foil served as a positron source, whereas ^60^Co was used for the acquisition of the prompt spectrum, i.e. the *quasi* instrument response function. Both types of measurements were carried out under the same conditions.

All necessary data and information regarding the data acquisition and data reduction are provided to allow reproducibility by other research groups.

**Specifications table**

**Table:** 

Subject area	Condensed matter physics, materials science, nuclear instrumentation
More specific subject area	Positron annihilation lifetime spectroscopy (PALS) on metals
Type of data	Tables, figures, text files
How data was acquired	Two-detector setup with plastic scintillators in co-linear (180°) arrangement using the DRS4 evaluation board for digitization (acquisition software: *DDRS4PALS* v1.04)
Data format	Raw spectra
Experimental factors	Lifetime spectrum acquisition:
	sample: tin (4N-Sn) annealed at 250 °C (5 h)
	source: ^22^Na (25μCi, 9.25×10^5^Bq) sealed in Kapton foil
	Prompt spectrum acquisition:
	source: ^60^Co (1μCi, 3.7×10^4^Bq)
Experimental features	The radioactive source ^22^Na was sandwiched between two identical tin samples (4N-Sn) (source-sample sandwich)
Data source location	University Wuerzburg, Department of Chemistry and Pharmacy, LCTM Roentgenring 11, D-97070 Wuerzburg
Data accessibility	Data is with this article
Related research article	N/A

**Value of the data**

• This completely transparent documentation of the experiment with respect to the setup, acquisition process and data reduction enables reproducibility by other research groups.
• Since reference values for specific bulk lifetimes of pure metals have a wide scatter in literature, the here provided experimentally obtained spectra can be taken to verify data acquired on pure tin samples.
• The here provided simulated spectra allow to verify the validity and functionality as well as the sensitivity and correctness of the algorithms (or software) used to decompose the relevant information, i.e. characteristic/specific lifetime and intensity.
• The simulated spectra allow to determine the maximum provided information content given by experimentally obtained spectra.
• The knowledge of the reference specific bulk lifetimes is crucial to obtain the correct source correction and, thus, to reduce artifacts in the following data treatment.

## 1 Data

The hereby provided data are divided into three collections, where collection 1 (ED1) and collection 2 (SD1) correspond to the experimentally obtained and simulated positron annihilation lifetime spectra of pure tin (4N-Sn), respectively. We use equivalent settings, which are shared in two files (*settingsExp.drs4LTSettings* and *settingsSim.drs4LTSettings*). Each collection ED1 and SD1 contains a set of six lifetime spectra with various statistics (500k, 1000k, 1500k, 2000k, 2500k and 2800k counts) acquired. The input of the simulated lifetime spectra (SD1) was derived from the retrieved specific lifetimes and its intensities of the experimentally obtained lifetime spectra (ED1) as an outcome of the least-square fitting procedure [2].

Furthermore, ED1 contains the data of the *quasi* instrument response function (IRF), i.e. the prompt spectrum, recorded using ^60^Co with an activity of 1μCi (3.7×10^4^Bq) (*specExp_co60.dat*). Additionally, the pulse height spectra (PHS) are provided. A third collection (Conf1) comprehends two setting files related to the type of data acquisition, i.e. the simulation or acquisition mode, using *DDRS4PALS* software [3]. The parameters most relevant for the timing determination using the constant fraction principle are equal for both, the acquisition of the experimentally obtained and simulated spectra. Nevertheless, the setting files are separated into experiment (*settingsExp.drs4LTSettings*) and simulation (*settingsSim.drs4LTSettings*). The simulation input file, used for the spectra simulation, is included in Conf1. A detailed overview of the data structure is given in Table 1.

**Table 1:** Listing of the provided data divided into three collections ED1, SD1 and Conf1.

**collection**	**name of file**	**brief description**
**ED1**	specExp_500k.dat	experimentally obtained lifetime spectrum (500k counts)
	specExp_1000k.dat	
	specExp_1500k.dat	
	specExp_2000k.dat	
	specExp_2500k.dat	
	specExp_2800k.dat	experimentally obtained lifetime spectrum (2800k counts)
	phs_A.dat	pulse height spectrum (PHS - detector A)
	phs_B.dat	pulse height spectrum (PHS - detector B)
	specExp_co60.dat	experimentally obtained prompt spectrum using ^60^Co
	phs_A_co60.dat	pulse height spectrum (PHS - detector A) using ^60^Co
	phs_B_co60.dat	pulse height spectrum (PHS - detector B) using ^60^Co
**SD1**	specSim_500k.dat	simulated lifetime spectrum (500k counts)
	specSim_1000k.dat	
	specSim_1500k.dat	
	specSim_2000k.dat	
	specSim_2500k.dat	
	specSim_2800k.dat	simulated lifetime spectrum (2800k counts)
**Conf1**	settingsExp.drs4LTSettings	experiment: settings file (*DDRS4PALS* software)
	settingsSim.drs4LTSettings	simulation: settings file (*DDRS4PALS* software)
	simulationSn.drs4SimulationInputFile	simulation input file (*DDRS4PALS* software)

## 2 Experimental design, materials and methods

### 2.1 Experimental setup

**Fig. 1:**
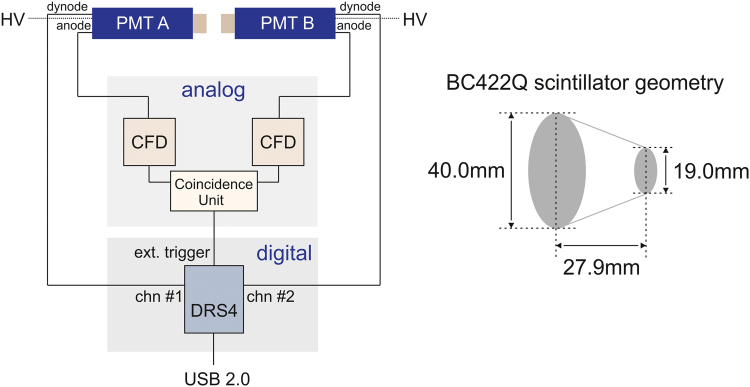
**Left:** Schematic illustration of the semi-digital/analog setup used for the acquisition of the lifetime and prompt spectra (Fig. 6). The analog section consists of two constant fraction discriminators (CFD) for each analog output of the photomultiplier tubes (PMT) A and B, and a coincidence unit, whose output serves as external trigger signal for the DRS4 evaluation board (digital section). The external trigger prompts the digitization of the dynode output pulses. **Right:** Geometry and dimensions of the conically shaped BC422Q (0.5% benzophenone) scintillator crystals, which are attached on the PMT׳s faceplate.

The positron lifetime measurements were carried out using the semi-digital/analog setup as shown in Fig. 1. The setup can be divided into the photo detection system (Section 1.1), an analog (Section 1.2) and a digital (Section 1.3) hardware section. Non-coincide events are pre-filtered by the analog section, whereas coincident events prompt the DRS4 evaluation board [1] (digital section) for digitization of the photomultiplier׳s dynode output pulses by an external trigger signal originated from the coincidence unit. Furthermore, the digitized pulses are processed using the software tool *DDRS4PALS* [3].

#### 2.1.1 Photo detection system

The photo detection system consists of two co-linearly (180°) arranged *Hamamatsu* (H1949-50/WA-5309) photomultiplier tubes (Fig. 1: PMT A and B) with conically shaped^1^ ultra-fast *BC422Q* (0.5 wt.% benzophenone) scintillators (Fig. 1, right). The PMTs A and B are separately power supplied by two high voltage units (Table 8), set to -2.09 kV and -2.11 kV, respectively. The scintillators are directly attached to the PMT faceplates using *Wacker* silicone oil (AK 10,000) to provide an optimal optical transition.

As displayed in Fig. 1, the anode outputs of the PMTs are connected to the constant fraction discriminators (CFD) of the analog section (1.2), whereas the dynode outputs are connected to the analog inputs of the DRS4 evaluation board (1.3).

#### 2.1.2 Acquisition hardware: analog section

As shown in Fig. 1, the analog section consists of two constant fraction discriminators (CFD), each one for the anode output of PMT A and B, and a coincidence unit (see Table 2 for specifications). The CFD windows were completely opened to pass all anode output pulses with the aim to emit a standard pulse immediately. Since both standard pulses of CFD A and B fall into the coincidence window of 2 μs, a rectangular shaped pulse is emitted by the coincidence unit and serves as external trigger signal for the DRS4 evaluation board to start the readout operation and digitization of the respective dynode pulses. Finally, the output signal of the coincidence unit is attenuated by -30 dB to fit the external trigger input range of the DRS4 evaluation board.

**Table 2:** Listing of the used analog hardware components regarding Fig. 1.

**analog: hardware component**	**type**	**supplier/manufacturer**
**constant fraction discriminator (CFD)**	CFD Diff Disc (583)	Ortec
**coincidence unit**	Universal Coincidence (418A)	Ortec
**high voltage (HV)**	High Voltage Supply (556)	Ortec
**photomultiplier tube (PMT)**	H1949-50/WA-5309	Hamamatsu
**scintillator crystal**	BC422Q (0.5 wt.% benzophenone)	Bicron/Saint-Gobain

#### 2.1.3 Acquisition hardware: digital section

Once the DRS4 evaluation board received the external trigger, the domino wave starts charging the switched capacitor arrays successively with the sampled voltages of the dynode output pulses (see principle of DRS4 chip [1]) using a sampling rate of 5.12 GHz with a sweep of 200 ns (Table 3). This leads to an average sampling increment of 195 ps for 1024 sampling points (capacitors). Subsequently, the stored pulses are digitized by an 80MHz ADC (14 bit) and read out via USB 2.0. Moreover, the pulses are attenuated by -20dB before connecting to the DRS4 evaluation board to fit the analog input range of ±500 mV. Before data acquisition, the DRS4 evaluation board was voltage and timing calibrated to reduce noise using the *DRSOsc* software [4].

**Table 3:** Listing of the used DRS4 evaluation board settings.

**digital: DRS4 evaluation board settings**	**value**
**sampling rate**	5.12 GHz
**number of cells/sweep**	1024 (200 ns)
**analog input range**	±500 mV
**trigger type**	external
**evaluation board type**	9
**firmware version**	21305

The evaluation board enables a convenient way of software-based pulse processing, as it was shown elsewhere by Petriska et al. [5] and Bin et al. [6].

### 2.2 Sample preparation

**Fig. 2:**
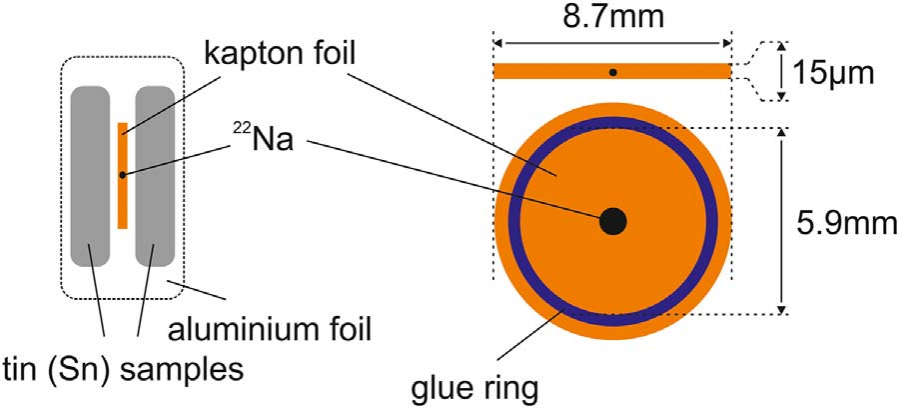
**Left:** Illustration of the source-sample sandwich. **Right:** Geometry of the positron source covered by Kapton foils.

Two tin (4N-Sn, Advent Research Materials Ltd.) plates of equivalent dimensions 10×10×1 mm were annealed at 250 °C for 5 h without vacuum or protective gas flow. Hence, the defect density (dislocations and vacancies) is reduced below the detection limit for positrons in metals [7] and the specific bulk lifetime outweighs. ^22^Na with an activity of 25μCi (9.25×10^5^Bq) serves as positron source and is covered by two Kapton foils with a combined thickness of approximately 15 μm (Fig. 2). Moreover, the Kapton foils are fixed by a thin ring of glue. For measurement, the positron source is centered between the tin samples, which is commonly described as source-sample sandwich. To avoid a displacement between the source and samples during the measurement, the sandwich was wrapped into household aluminium foil.

### 2.3 Acquisition of lifetime spectra

The hereby provided positron lifetime spectra (Table 1), were obtained using the software tool *DDRS4PALS* v1.04 [3]. The channel width (binning) was chosen to be 5 ps (10,000 channels/bins upon 50 ns).

*DDRS4PALS* can be executed in two *modi*, the *acquisition* and the *simulation* mode. The *acquisition* mode processes the dynode output pulses, recorded and digitized by the DRS4 evaluation board, whereas the *simulation* mode is based on the C++ library *DLTPulseGenerator* [8], [9], [10] which provides the simulation of lifetime spectra according to the measurement setup.

However, for both types of lifetime spectra, i.e. the experimental and the simulated, the equivalent parameter values, as stored in the setting files *settingsExp.drs4LTSettings* and *settingsSim.drs4LTSettings*, were applied. The parameters, most relevant for the timing determination (Section 3.1) and the pulse height discrimination (Section 3.2), are listed in Table 4, Table 5.

**Table 4:** Listing of the parameters, most relevant for the timing determination.

**pulse timing determination**	**value**
**signal output**	dynode
**pulse rise time**	2.5 ns
**d-CFD level A**	25%
**d-CFD level B**	25%
**algorithm type for d-CFD**	cubic spline interpolation (ALGLIB [11])

**Table 5:** Listing of the pulse height spectra (PHS) windows of detector A and B used for the energy selection of start and stop events (see coloured frames in Fig. 4).

**pulse height spectrum (PHS): A**	**value**
**start (LL)**	230 mV
**start (UL)**	454 mV
**stop (LL)**	78 mV
**stop (UL)**	107 mv

**pulse height spectrum (PHS): B**	**value**

**start (LL)**	231 mV
**start (UL)**	466 mV
**stop (LL)**	84 mV
**stop (UL)**	116 mV

#### 2.3.1 Pulse timing

**Fig. 3:**
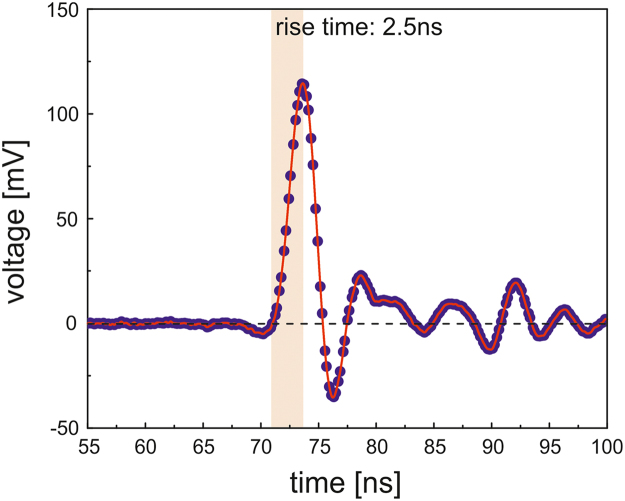
Digitized PMT (Hamamatsu of type H1949-50/WA-5309) dynode output pulse (blue dots): The red line indicates the cubic spline interpolated output pulse, whereas the orange shaded frame represents the region relevant for the timing determination using the software-based constant fraction principle (d-CFD). The rise-time of the dynode output pulses is approximately 2.5 ns. No prior corrections on the baseline were applied.

The software-based constant fraction principle (d-CFD) was accomplished to determine the correct timing of the digitized dynode output pulses using a d-CFD level of 25% for both branches A and B. A cubic spline interpolation (ALGLIB [11]) of the sample points was applied before the d-CFD determination to provide a smooth pulse shape. The d-CFD value was calculated from the spline interpolated maximum (amplitude) without applying prior corrections on the baseline. The rising edge includes approximately ten sampling points, as displayed in the orange shaded region in Fig. 3.

For the simulated lifetime spectra, the rise-time of the modelled detector output pulses was set to 2.5 ns (see file *simulationSn.drs4SimulationInputFile*), as this is the rise-time of the dynode output pulses (Fig. 3).

#### 2.3.2 Pulse height discrimination

**Fig. 4:**
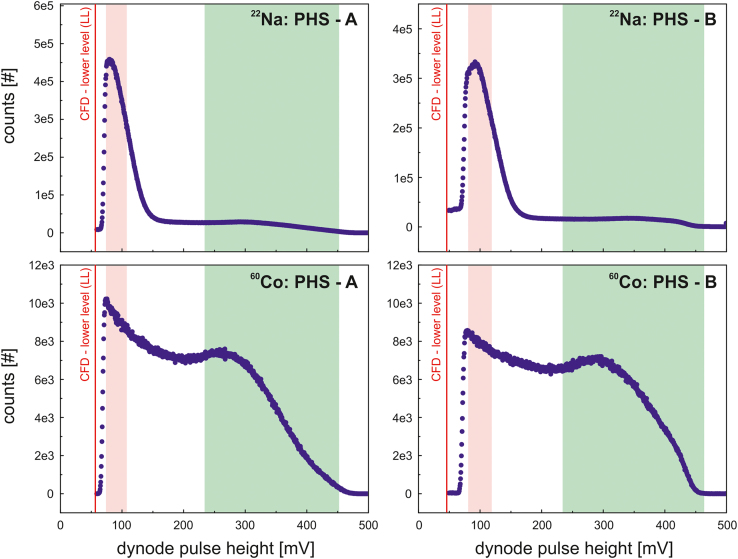
Pulse height spectra (PHS) of scintillators BC422Q (0.5% benzophenone) for ^22^Na (top) and ^60^Co (bottom). The coloured frames indicate the energy windows of the start (green) and stop (red) events. The red line represents the lower level (LL) of the constant fraction discriminator in the analog section (CFD, Fig. 1) and specifies the minimum accepted amplitude (red line).

The PHS windows, as specified in Table 5, of detector A and B (Fig. 1) are used for the energy selection of start (Fig. 4, green frame) and stop (Fig. 4, red frame) events. They are identical for the acquisition of the lifetime and prompt spectra.

To reduce the data transfer rate through the USB 2.0^2^ interface and, hence, preventing the digitization of negligible events, the lower level (LL) of the CFD window was shifted to pulse heights (amplitude) close to the LL of the stop window (Fig. 4, red frame) in the PHS, as it is indicated by the red line in Fig. 4. Further performance improvements can be achieved by using only one combination of start and stop branch (e.g. A as start and B as stop). Therefore, the digitization can be significantly reduced by adapting the lower (LL) and upper level (UL) of the CFD windows to the relevant pulse heights (amplitude) in the PHS only.

**Fig. 5:**
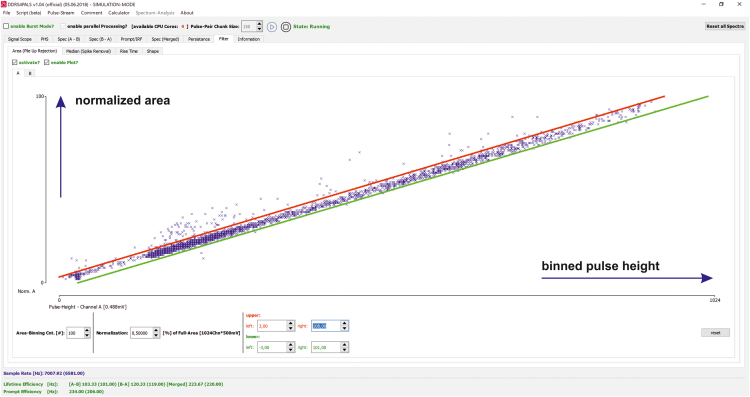
Screenshot of software DDRS4PALS v1.04 [3] showing the upper (red) and lower (green) levels of the applied area filter of branch A, measuring the tin samples with a ^22^Na positron source. Only pulse areas within the bounding are considered for the further lifetime calculation. The normalized area is displayed as a function of the pulse height. Further filters, such as median (spike removal) or pulse-shape can be applied but were not considered in this work.

However, the PHS, as shown in Fig. 5, are related to the files *phs_A.dat/phs_B.dat* and *phs_A_co60.dat/phs_B_co60.dat*.

#### 2.3.3 Pulse filtering: area filter

**Fig. 6:**
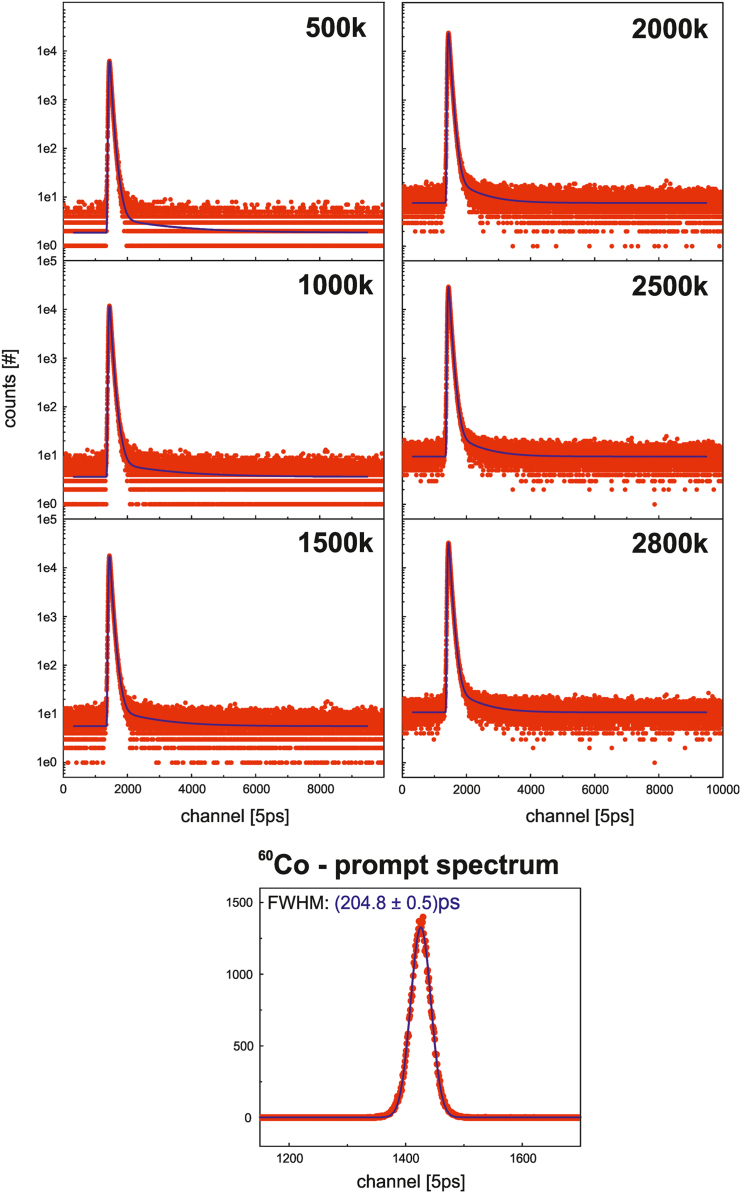
Experimentally obtained positron lifetime spectra (red dots) for different statistics acquired measuring pure tin (4N-Sn). The number of used channels is 10,000 with a channel width of 5 ps. Moreover, the blue line indicates the best fit. The *quasi* instrument response function (IRF – prompt spectrum) was measured using ^60^Co (1μCi). Subsequently, a FWHM of (204.8±0.5)ps was determined using a single Gaussian fit as guide for the eye.

Pile-up events were rejected by applying a pulse area filter on both branches A and B, as shown in Fig. 5, which essentially leads to an improved spectra quality. For the measurement of the *quasi* instrument response function (IRF) using ^60^Co (Fig. 6), the same windows were applied (see file *settingsExp.drs4LTSettings*).

### 2.4 Lifetime spectra analysis

The experimentally obtained and simulated lifetime spectra were analysed (Table 8, Table 9, Table 11, Table 12) using the software tool *DQuickLTFit* v4.0 [12]. The employed fit model is the commonly used analytical solution of the convolution of a sum of N exponential distributions with a sum of gaussian distribution functions, as first published by Kirkegaard et al. [2]. Moreover, *DQuickLTFit* has implemented the *MPFIT* library [13] for solving the non-linear least-square problem using the *Levenberg-Marquardt* algorithm [14], [15]. The analysis was performed on

(1) the initial lifetime spectra, acquired with a channel width of 5 ps (bin factor: 1, Table 8, Table 11), and

**Table 8:** Retrieved relevant parameters of the experimentally obtained lifetime spectra with bin factor: 1 (channel width: 5 ps): the specific lifetimes τ_i_ and corresponding intensities I_i_, and the FWHM, contributions c and relative shifts Δt to t_0_ = 5468ps of the modelled Gaussian IRFs (G_1_/G_2_) at different counts acquired using a least-square fit. The bracketed values indicate the standard errors of the fitted values.

**acquired counts**	**τ**_**1**_**[ps]**	**I**_**1**_	**τ**_**2**_**[ps]**	**I**_**2**_	**τ**_**3**_**[ps]**	**I**_**3**_
500k	**196.9**	**0.862**	423	0.1337	3904	0.00470
	(3.8)	(0.029)	(22)	(0.0029)	(559)	(0.00040)
1000k	**197.0**	**0.864**	429	0.1312	4535	0.00520
	(2.7)	(0.019)	(15)	(0.0019)	(414)	(0.00030)
1500k	**194.3**	**0.842**	405	0.1530	3658	0.00530
	(2.7)	(0.020)	(13)	(0.0024)	(270)	(0.00030)
2000k	**194.6**	**0.837**	399	0.1570	3269	0.00570
	(2.4)	(0.019)	(11)	(0.0023)	(202)	(0.00020)
2500k	**192.6**	**0.8236**	387	0.1706	3053	0.00580
	(2.5)	(0.0191)	(10)	(0.0026)	(170)	(0.00020)
2800k	**191.9**	**0.8192**	384.9	0.1750	3062	0.00580
	(2.4)	(0.0182)	(9.5)	(0.0026)	(158)	(0.00020)

	**G**_**1**_**: FWHM [ps]**	**G**_**1**_**: c**	**G**_**1**_**: Δt [ps]**	**G**_**2**_**: FWHM [ps]**	**G**_**2**_**: c**	**G**_**2**_**: Δt [ps]**

500k	**192.6**	**0.899**	2.64	**301**	**0.101**	−21
	(2.8)	(0.040)	(0.89)	(13)	(0.040)	(25)
1000k	**195.4**	**0.922**	3.14	**311**	**0.0783**	−23
	(1.8)	(0.027)	(0.60)	(12)	(0.027)	(24)
1500k	**197.6**	**0.937**	1.15	**328**	**0.063**	−23
	(1.3)	(0.019)	(0.44)	(13)	(0.019)	(25)
2000k	**198.1**	**0.945**	0.81	**317**	**0.055**	−40
	(1.3)	(0.021)	(0.42)	(15)	(0.021)	(33)
2500k	**198.2**	**0.942**	0.80	**326**	**0.058**	−31
	(1.1)	(0.017)	(0.35)	(12)	(0.017)	(24)
2800k	**198.5**	**0.944**	0.83	**328**	**0.057**	−30
	(1.0)	(0.015)	(0.33)	(11)	(0.015)	(23)

**Table 11:** Simulated lifetime spectra with bin factor: 1 (channel width: 5 ps): Retrieved specific lifetimes and its corresponding intensities for different counts acquired using a least-square fit. The bracketed values indicate the standard errors of the fitted values.

**acquired counts**	**τ**_**1**_**[ps]**	**I**_**1**_	**τ**_**2**_**[ps]**	**I**_**2**_	**τ**_**3**_**[ps]**	**I**_**3**_	**FWHM [ps]**
500k	**194.6**	**0.836**	395	0.1607	2954	0.0036	**204.27**
	(2.8)	(0.028)	(18)	(0.0036)	(528)	(0.0003)	(0.71)
1000k	**192.4**	**0.824**	388	0.1713	2858	0.0046	**204.91**
	(2.0)	(0.020)	(12)	(0.0028)	(259)	(0.0003)	(0.50)
1500k	**192.7**	**0.824**	387.5	0.1715	3083	0.0049	**205.07**
	(1.7)	(0.016)	(9.3)	(0.0023)	(207)	(0.0003)	(0.41)
2000k	**192.3**	**0.820**	385.1	0.1751	3096	0.0049	**205.21**
	(1.4)	(0.014)	(8.0)	(0.0021)	(175)	(0.0003)	(0.36)
2500k	**191.5**	**0.815**	381.1	0.1804	3022	0.0050	**205.42**
	(1.3)	(0.013)	(7.1)	(0.0020)	(151)	(0.0003)	(0.32)
2800k	**190.3**	**0.804**	374.0	0.1907	2883	0.0052	**205.58**
	(1.3)	(0.013)	(7.6)	(0.0021)	(133)	(0.0003)	(0.31)
(2) the re-binned lifetime spectra, by applying a bin-factor of ten, which results in a channel width of 50 ps (bin factor: 10, Table 9, Table 12).

**Table 9:** Retrieved relevant parameters of the experimentally obtained lifetime spectra with bin factor: 10 (channel width: 50 ps): the specific lifetimes τ_i_ and corresponding intensities I_i_, and the FWHM, contributions c and relative shifts Δt to t_0_ = 5468 ps of the modelled Gaussian IRFs (G_1_/G_2_) at different counts acquired using a least-square fit. The bracketed values indicate the standard errors of the fitted values.

**acquired counts**	**τ**_**1**_**[ps]**	**I**_**1**_	**τ**_**2**_**[ps]**	**I**_**2**_	**τ**_**3**_**[ps]**	**I**_**3**_
500k	**194.0**	**0.841**	406	0.1522	4083	0.00550
	(4.6)	(0.025)	(21)	(0.0039)	(474)	(0.00040)
1000k	**196.3**	**0.858**	424	0.1362	4343	0.00530
	(2.9)	(0.015)	(15)	(0.0021)	(391)	(0.00030)
1500k	**191.9**	**0.824**	389	0.1706	3416	0.00570
	(3.1)	(0.023)	(13)	(0.0032)	(237)	(0.00030)
2000k	**193.7**	**0.831**	393	0.1632	3209	0.00580
	(2.5)	(0.020)	(11)	(0.0026)	(194)	(0.00030)
2500k	**191.9**	**0.818**	384	0.175	3051	0.00590
	(2.5)	(0.016)	(10)	(0.016)	(167)	(0.00020)
2800k	**191.5**	**0.817**	383.4	0.1777	3056	0.00590
	(2.4)	(0.018)	(9.5)	(0.0027)	(159)	(0.00030)

	**G**_**1**_**: FWHM [ps]**	**G**_**1**_**: c**	**G**_**1**_**: Δt [ps]**	**G**_**2**_**: FWHM [ps]**	**G**_**2**_**: c**	**G**_**2**_**: Δt [ps]**

500k	**195.5**	**0.929**	−2.59	**332**	**0.071**	−24
	(2.2)	(0.031)	(0.71)	(19)	(0.031)	(36)
1000k	**196.7**	**0.936**	−1.81	**321**	0.064	−34
	(1.8)	(0.026)	(0.58)	(16)	(0.026)	(32)
1500k	**197.7**	**0.933**	−3.75	**332**	**0.067**	−21
	(1.3)	(0.019)	(0.43)	(12)	(0.019)	(22)
2000k	**198.1**	**0.944**	−4.19	**320**	**0.057**	−41
	(1.3)	(0.021)	(0.42)	(14)	(0.021)	(31)
2500k	**198.4**	**0.941**	−4.16	**327**	**0.059**	−34
	(1.1)	(0.017)	(0.36)	(12)	(0.017)	(24)
2800k	**198.6**	**0.943**	−4.17	**330**	**0.057**	−34
	(1.0)	(0.015)	(0.33)	(11)	(0.015)	(23)

**Table 12:** Simulated lifetime spectra with bin factor: 10 (channel width: 50 ps): Retrieved specific lifetimes and its corresponding intensities for different counts acquired using a least-square fit. The bracketed values indicate the standard errors of the fitted values.

**acquired counts**	**τ**_**1**_**[ps]**	**I**_**1**_	**τ**_**2**_**[ps]**	**I**_**2**_	**τ**_**3**_**[ps]**	**I**_**3**_	**FWHM [ps]**
500k	**195.2**	**0.841**	402	0.1547	3500	0.00430	**204.36**
	(2.6)	(0.025)	(16)	(0.0030)	(440)	(0.00030)	(0.72)
1000k	**192.6**	**0.826**	390	0.1695	3090	0.00480	**204.90**
	(1.9)	(0.019)	(11)	(0.0026)	(254)	(0.00030)	(0.51)
1500k	**192.8**	**0.826**	389.5	0.1696	3172	0.00490	**205.11**
	(1.6)	(0.016)	(9.2)	(0.0022)	(208)	(0.00030)	(0.42)
2000k	**192.4**	**0.821**	385.4	0.1745	3083	0.00500	**205.20**
	(1.4)	(0.015)	(8.0)	(0.0021)	(173)	(0.00030)	(0.37)
2500k	**191.8**	**0.817**	382.9	0.1782	3077	0.00500	**205.37**
	(1.3)	(0.013)	(7.1)	(0.0019)	(154)	(0.00030)	(0.33)
2800k	**190.5**	**0.806**	375.0	0.1893	2961	0.00520	**205.54**
	(1.3)	(0.013)	(6.6)	(0.0021)	(136)	(0.00030)	(0.31)

#### 2.4.1 Experimentally obtained lifetime spectra

For the spectra analysis in high-purity, well annealed samples, three specific lifetimes, originating from

• the bulk material (in this case tin, 4N-Sn),
• the ^22^Na salt and the Kapton foil (combined),
• and the formed positronium

are expected to be extracted from the experimentally obtained lifetime spectra. The fitting parameters and start values of the expected specific lifetimes and its corresponding intensities are listed in Table 6, Table 7 and served for the analysis/fitting of all lifetime spectra acquired on various statistics (see Fig. 6). No restrictions on the parameters, i.e. fixed values or upper/lower limits, were made for the non-linear least-square fitting procedure.

**Table 6:** Parameters used for the least-square fitting procedure of the experimentally obtained and simulated lifetime spectra. The fit was applied on the lifetime spectra for the binning factors one and ten which results in a channel width of 5 ps and 50 ps, respectively.

**bin factor: 1 (channel width: 5** **ps)**	**least-square fitting parameters**
**ROI [start:stop]**	[329:9480]
**channel region: background calculation**	[7481:9480] = 2000
**fixed background?**	no
**fit weighting**	w_i_ = y_i_^-1/2^

**bin factor: 10 (channel width: 50** **ps)**	**least-square fitting parameters**

**ROI [start:stop]**	[33:948]
**channel region: background calculation**	[749:948] = 200
**fixed background?**	no
**fit weighting**	w_i_ = y_i_^-1/2^

**Table 7:** Start values of the least-square fitting procedure used for determination of the specific lifetimes and its intensities as well as the instrument response function (IRF) using a superposition of two Gaussian functions (G_1_/G_2_). The start values for the specific lifetimes and corresponding intensities were used for both types of lifetime spectra: the experimentally obtained and the simulated. The shortest component (τ_1_/I_1_) relates to the bulk (tin), whereas the other contributions, i.e. τ_2_/I_2_ and τ_3_/I_3_, relate to the Kapton foil and ^22^Na (salt) in combination, and positronium, which is probably forming at interfaces between source and source covering foil (see Fig. 2).

	**value**
**τ**_**1**_**[ps]**	**198**
**I**_**1**_	0.900
**τ**_**2**_**[ps]**	**380**
**I**_**2**_	0.100
**τ**_**3**_**[ps]**	**2200**
**I**_**3**_	0.005
**G**_**1**_**: FWHM [ps]**	**198**
**G**_**1**_**: I**_**1**_	0.5
**G**_**2**_**: FWHM [ps]**	**198**
**G**_**2**_**: I**_**2**_	0.5

#### 2.4.2 Simulated lifetime spectra

The retrieved specific lifetimes and its corresponding intensities of the experimentally obtained lifetime spectrum with the highest statistics (2.8 million counts, Table 8) acquired, served as base for the simulation input (Table 10). The instrumental response (IRF) was given using a single Gaussian, where the FWHM corresponds to the *effective* FWHM of the superposed two Gaussian IRFs (G_1_/G_2_ – see Table 8, 2800 k) under the condition that their relative shifts Δt = 0 ps. Then, the resulting simulated FWHM was determined following the instruction as shown in [8]: Assuming a completely symmetric setup, the FWHM for the *PDS A/B* were chosen to be 146 ps, whereas the FWHM of the *MU* is 2.5 ps (see file *simulationSn.drs4SimulationInput*).

**Table 10:** Specific lifetimes and its intensities, which served as simulation input (see file*simulationSn.drs4SimulationInput*). As the instrumental response (IRF), a single Gaussian with a FWHM corresponding to the *effective* FWHM (condition: Δt = 0 ps) of the experimentally obtained spectrum with the highest statistics (2.8 million counts, Table 8) acquired, was taken.

**simulation input**	**value**
**τ**_**1**_**[ps]**	192
**I**_**1**_	0.82
**τ**_**2**_**[ps]**	385
**I**_**2**_	0.175
**τ**_**3**_**[ps]**	3050
**I**_**3**_	0.005
**FWHM [ps]**	205

## Footnotes

1 The conically shaped scintillators allow a close arrangement of multiple detectors around the source-sample sandwich (Section 2).

2 The data transfer rate of USB 2.0 is limited to 25 Mbyte/s. The data size of each digitized pulse pair (detector A and B) is 16 kByte = 1024 (readout cells)×2 (time and voltage of A)×2 (time and voltage of B)×4 (size of data type *float* in bytes). Therefore, the efficiency is limited to approximately 500 pulse pairs/s (see also [5]).
