# Supplementary material for: Data on pure tin by Positron Annihilation Lifetime Spectroscopy (PALS) acquired with a semi-analog/digital setup using DDRS4PALS
Source: Data Brief. 2018 Nov 28;22:16–29. doi: 10.1016/j.dib.2018.11.121 (PMC6293023; doi:10.1016/j.dib.2018.11.121)
Supplement: Supplementary file 1 — Supplementary material. [file mmc1.pdf]

Conflicts of Interest Statement

Manuscript title: Data on pure tin by Positron Annihilation Lifetime Spectroscopy (PALS) acquired with a semi-analog/digital setup using DDRS4PALS

The authors whose names are listed immediately below certify that they have NO affi liations with or involvement in any organization or entity with any fi nancial interest (such as honoraria; educational grants; participation in speakers' bureaus; membership, employment, consultancies, stock ownership, or other equity interest; and expert testimony or patent-licensing arrangements), or non-fi nancial interest (such as personal or professional relationships, affi liations, knowledge or beliefs) in the subject matter or materials discussed in this manuscript.

Author names:

Danny Petschke,
Ricardo Helm,
Dr. habil. Torsten Staab

The authors whose names are listed immediately below report the following details of affiliation or involvement in an organization or entity with a financial or non-financial interest in the subject matter or materials discussed in this manuscript. Please specify the nature of the conflict on a separate sheet of paper if the space below is inadequate.

Author names:

This statement is signed by all the authors to indicate agreement that the above information is true and correct (*a photocopy of this form may be used if there are more than 10 authors*):

Author's name (typed)	Author's signature	Date
Danny Petschke		06.11.2018
Ricardo Helm		06.11.2018
Dr. habil. Torsten Staab		06.11.2018
